# Supplementary material for: Venomix: a simple bioinformatic pipeline for identifying and characterizing toxin gene candidates from transcriptomic data
Source: PeerJ. 2018 Jul 31;6:e5361. doi: 10.7717/peerj.5361 (PMC6074769; doi:10.7717/peerj.5361)
Supplement: Supplemental Information 3 [file peerj-06-5361-s003.gz › FinalOutput_E-6/Toxins/Snake_venom_metalloprotease_inhibitor_02A10_1/finaltree.pdf]

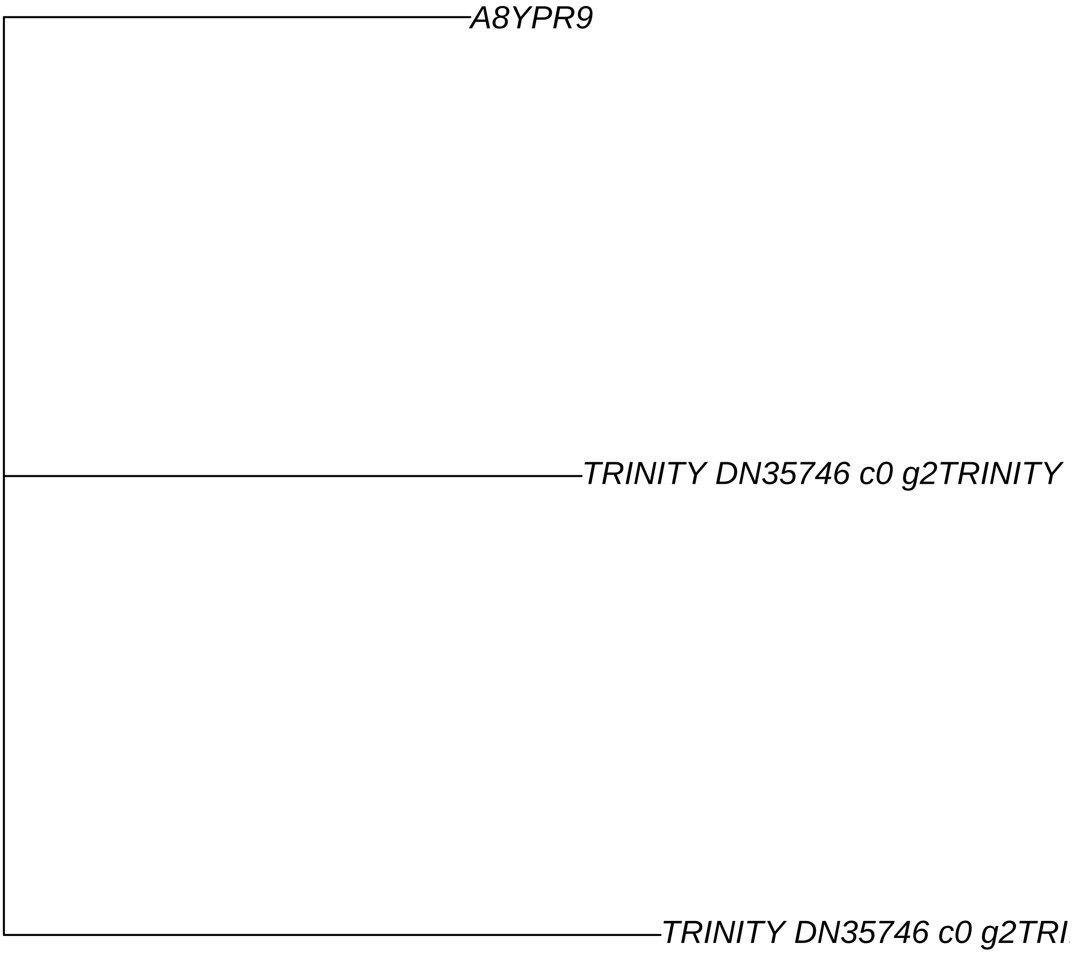
A8YPR9
TRINITY DN35746 c0 g2TRINITY
TRINITY DN35746 c0 g2TRI
